# Ligation of the Dorsal Vein of the Penis as a Cure for Atonic Impotence*Read at the December meeting of the Austin District Medical Society.

**Published:** 1903-02

**Authors:** Joe S. Wooten

**Affiliations:** Austin, Texas


For Texas Medical Journal.

### Ligation of the Dorsal Vein of the Penis as a Cure for Atonic Impotence.*

BY JOE S. WOOTEN, B. S., M. D., AUSTIN, TEXAS.

The normal physiological process of erection of the penis is produced by excitation of both brain and spinal cord centers. It may also be produced by impressions originating outside of the nerve centers or any portion of the nervous system. The spinal cord centers are probably located in the cervical and sacral portions of the cord. An erection is induced by one of three ways: (1) By an increased influx of blood to the organ; (2) by a diminished efflux of blood from the organ; (3) or by a combination of both of these conditions. The first of these conditions, the increased flow of blood, is produced either by stimulation of the nerve centers or from peripheral irritation of a nerve. The second condition, that of congestion, is the result of mechanical compression by certain muscles, assisted by the peculiar anatomical arrangement by which the veins at the base of the penis twist at their points of exit from the corpora cavernosa and spongiosum. There comes about first, a relaxation of the muscular fibres in the trabeclae of the corpora

*Read at the December meeting of the Austin District Medical Society.

and spongiosum and in the arteries of these parts, as a result of which there takes place an influx of blood; the rigidity of the penis immediately takes place as soon as this relaxation of the muscular fibres is complete and the venous sinuses are filled. The check to the return of the blood through the veins, which are much larger in calibre than the arteries, and which would otherwise empty themselves much faster than the arteries could possibly supply blood, is maintained by the action of the bulbo-cavernosi, the ischio-cavernosi and the adductor prostate, upon the deep and superficial veins of the penis. That such a physiological process takes place and is the most probable explanation of the power of erection, has been satisfactorily confirmed by experiments upon the dog and derived from observations made upon man in disease.

By atonic impotence we mean a failure in satisfactory coitus, whether such failure manifests itself in precipitate emission, delayed emission, incomplete erection or absence of sexual desire. The disturbance with emission and erection are principal types of the disease for which we are most consulted, the last named condition, absence of sexual desire, being usually an advanced or later stage of complete exhaustion of this disease. To enter into a discussion of the pathology and etiology of sexual impotence would take me wide of the particular phase of the subject which I desire to present for your consideration. The underlying cause in all these conditions, be the primary cause what it may, is impaired nervous function and stimulus due to exhaustion of specialized nerve tracts and centers in brain and spinal cord, by which the sexual function is torpid or temporarily suspended. Ligation of the dorsal vein of the penis is not a cure for every case of atonic impotence, and it is only in a few selected cases of the vast number of afflicted patients who present themselves for treatment where you will find such surgical resort necessary or conditions indicating such a procedure. The accepted and well established practice of hunting for and removing all local inflammations and focal lesions in and about the genito-urinary tract, together with the rational use of medical, mechanical, moral and suggestive therapeutics, will still have their prominent place in the treatment of this condition, but after all possible reflex sources of trouble have been removed and these remedies applied and you have failed to restore a normal erection and a satisfactory coitus, which you will often do, it is then that I advocate the ligation of the dorsal vein of the penis, and believe its field of usefulness will be found wide in perfecting a cure. It does it in this way: Some of you are doubtless familiar with a few of the mechanical devices designed to assist in bringing about a firm erection in an otherwise feeble organ, one of which I here exhibit. Its mechanism is simple and its therapeutic use,. while questionable upon ethical grounds, is nevertheless based upon sound sense and mechanics. It performs its function by compressing the veins and preventing their too hurried emptying, thus maintaining erection. In atonic impotence there is a loss of tonicity in all of the tissues, and a relaxed, dilated condition of the veins and sinuses. The ligation of the dorsal vein cuts off the main exit of venous blood and collateral circulation eventually takes its place.

In case of the patient upon whom I tried this operation as advised and recommended by Broome, Murry and others, I had treated for hyperesthesia of the prostatic urethra and acute seminal vesiculitis, attended by nocturnal pollutions, the combined effect of the habit of masturbation. All local conditions were cured, the myelasthenia relieved and the patient's general health restored. His urethral calibre was 27 French. His normal weight remained at 190 pounds. While sexual intercourse and sexual excitement were interdicted, as it should be in every case of sexual impotency, the patient persisted in ocasionally trying, only to result in partial or complete failure, emission always taking place. This period of impotency lasted altogether for about three years. Repeated suggestion was practiced, moral advice given, and finally resort to sleeping with a female companion—without attempt at coitus,—so as to overcome any psychical influence, was tried (partly at my suggestion), but with little satisfactory results. While in the metropolis of the East, he consulted an eminent genito-urinary specialist, who—as a last resort to do something—cut an imaginary stricture to 32 French sound. Several months after his return home, he again consulted me in reference to his case, and I then advised operative treatment, believing that the partial and incomplete erection, with precipitate emission, were the result of a too rapid emptying of the sinuses of the corpora and spongiosum. Open ligation with the cat-gut was done. For the first twenty-four or thirty-six hours the patient complained bitterly from painful erection, which was almost constant. Attempt at intercourse was prohibited for a period of two months, and the patient cautioned against its practice. It has been now about four months since the operation, and the party reported to me not long since that he had had for the first time in nearly three years complete and satisfactory coitus and was now willing to stop trying.

AUTHORITIES.

Diseases of the Male and Female Sexual Organs, Taylor.
Diseases of the Male Sexual Organs, Gross.
Sexual Debility, Sturgis.
Quain's Anatomy.
Anatomy, by Gerrish.

